# Investigation of benzoic acid and sorbic acid concentrations in tomato paste, pepper paste, ketchup, mayonnaise, and barbeque sauce samples by headspace gas chromatography-mass spectrometry

**DOI:** 10.55730/1300-0527.3663

**Published:** 2024-01-22

**Authors:** Orhan DESTANOĞLU

**Affiliations:** Department of Science, Institute of Forensic Sciences and Legal Medicine, İstanbul University-Cerrahpasa, İstanbul, Turkiye

**Keywords:** Benzoic acid, sorbic acid, sauces, tomato paste, food quality control, food safety

## Abstract

In this study, it was aimed at investigating benzoic acid (BA) and sorbic acid (SoA) concentrations in tomato paste, pepper paste, ketchup, mayonnaise, and barbeque sauce samples by a validated static headspace gas chromatography–mass spectrometry (GC-MS) method. Salicylic acid (SalA) was used as internal standard and the measurements were conducted in the wide linear concentration ranges of BA and SoA which were 2.5–5000 and 12.5–5000, respectively. The limit of detections (LODs) were determined to be 1.5 and 4.5 mg/kg while the limit of quantifications (LOQs) were 2.5 and 12.5 mg/kg for BA and SoA, respectively. The average recovery% values of BA and SoA were found to be 98.5% and 98.7% in an open tomato paste sample while these values were 98.7% and 100.3% in a mayonnaise sample, respectively. Accuracy of the proposed method was confirmed by statistically (significance test) evaluating excellent recovery values. In real samples, while the results of the canned tomato pastes and industrial sauce samples were found suitable, BA and SoA ​​were determined in some tomato and pepper paste products sold under the traditional or homemade name although use of the preservatives in the pastes were prohibited. It is vital for public health to prevent adulteration in pastes which is indispensable for Turkish cuisine as well as prevalently consumed in the world. Therefore, the proposed method can be used in food control laboratories due to its reliability and consumption of much less toxic chemical reagents.

## 1. Introduction

Food preservatives are chemicals added to some foods to extend shelf life span and while maintaining the quality, freshness, and taste of them. Benzoic acid (benzenecarboxylic acid, BA), sorbic acid (2,4-hexanedienoic acid, SoA), and the salts derived from them are common and cheap food additives which effectively inhibit the proliferation of the microorganisms such as fungi, yeasts, moulds, and bacteria [1–8]. The ingested amount of these chemicals elevated in direct proportion to the increase in the use of preservatives in foods. In every country, there is an upper limit of BA and SoA concentrations that changes from country to country, but in general, they are between 0.15–6 g/kg, according to the type of food they are added to, unless their use are prohibited [9]. It is commonly admitted that the use of BA and/or SoA below the maximum permitted limits do not affect health, but excess intake of these chemicals can lead to metabolic acidosis, genotoxicity, convulsions, asthma, urticaria, and hyperpnoea [1–8].

Consumption of foods in which tomatoes and peppers are used as main ingredients, especially pastes, is quite common in Türkiye. The Turkish Food Codex Communiqué on Paste and Similar Products states that the use of sorbic acid (E 200) and potassium sorbate (E 202) in the tomato and pepper paste products is not allowed. The Turkish Food Codex Regulation on food additives complies with the Regulation (EC) No 1333/2008 of the European Parliament and of the Council on food additives [10,11]. Moreover, it is also specified by the Communiqué that the tomato/pepper-based brands that give the impression of tomato or pepper paste on the label of edible products, paste image, tomato/pepper image and similar expressions and visuals must not be used in the similar products. However, some people aimed to prevent the perception of tomato/pepper-based edible products as paste and caused an unfair competition. On the other hand, for the ketchup, mayonnaise, and barbeque sauce products consumed by people of all ages, it is allowed to use certain concentration limits of preservatives according to the regulation, depending on whether they are emulsified or not. The concentration levels of BA and SoA in these widely consumed products should be monitored strictly for food safety. Hence, there is a need for a method that not only is easy and reliable, but also generates smaller amount of toxic chemical waste, which is crucial for environmental sustainability today.

A number of studies have been published in the literature for determination of the analytes we are interested in, on various types of food, by employing some analytical instruments, such as headspace solid-phase microextraction-gas chromatography-flame ionization detector (HS-SPME-GC-FID) [12], GC-MS analysis after SPME [13], HS-GC-MS [3,14], high-performance liquid chromatography (HPLC) [4,8,15–22], thermal desorption gas chromatography mass spectrometry (TD-GC-MS) [23], capillary zone electrophoresis (CZE) [6,7,24], CE [2,25], capillary ion chromatography with conductivity detection (CIC-CD) [5], paper spray mass spectrometry (PS-MS) [26], and quantitative proton nuclear magnetic resonance (qHNMR) spectroscopy [27]. Among them, determining BA and SoA in a simultaneous manner has most commonly studied with HPLC, but it involves some drawbacks, for example, mobile phases need copious amounts of organic solvents and HPLC requires tedious processes regarding the pretreatment of the sample. Most of the foodstuff possess complex matrices and thus, some clean-up processes generally required prior to chromatographic analyses such as solid phase extraction (SPE), dispersive liquid-liquid microextraction (DLLME), liquid-liquid extraction (LLE), and QuEChERS methods to eliminate the coextracted matrix interferences [4,5,12,15,20,21].

In this work, it was aimed to simultaneously analyze BA and SoA in tomato paste, pepper paste, ketchup, mayonnaise, and barbeque sauce samples by a fast, easy, reliable and sensitive static HS-GC-MS method with using and wasting minimal volumes of chemical reagents. A static HS-GC-MS was utilized with the parameters developed in the previously published study [14] for nonalcoholic beverages, but merely a minor modification on sample preparation step was applied prior to transferring the sample to the HS vial in where the analytes and the internal standard (IS, salicylic acid, SalA) were simultaneously derivatized to their more volatile methyl esters. It was proven by the results obtained from the validation studies that the method worked successfully in this study. The remarkable advantage of the method was that even though a very small amount of the samples and the reagents (methanol and sulfuric acid) employed for the derivatization reaction were used, very sensitive and reliable analyses were achieved.

The obtained results were evaluated according to The Turkish Food Codex Regulation on Food Additives [11] and the Turkish Food Codex Communiqué on Paste and Similar Products [28]. BA and SoA content in canned and known brands’ products have been found to comply with the regulations. Contrary to the industrial branded products, tomato and pepper pastes sold openly under the traditional image and home-made name were in violation of the regulation in that both preservatives were determined. Noteworthy is that to the best of the author’s knowledge, this is the first study to analyse BA and SoA in tomato paste, pepper paste, ketchup, mayonnaise, and barbeque sauce samples by performing a static HS-GC-MS. The author claims that the proposed method is a candidate for determination of BA and SoA in different kinds of sauces for both quality control analyses and finding out adulterations.

## 2. Experimental

### 2.1. Chemicals and reagents

Sorbic acid (≥ 99.0%), benzoic acid (ACS reagent, ≥ 99.5%), salicylic acid (ACS reagent, ≥ 99.5%), and methanol (HPLC grade, ≥ 99.9%) were procured from Sigma-Aldrich. Sulfuric acid (analytical reagent grade, ≥ 95.0%) was purchased from Fischer Scientific UK. Ultra pure water (≥ 18.2 MΩ cm resistivity) was obtained from a New Human Power I Scholar UV water purification system (Human Corporation, Seoul, Korea).

### 2.2. Solutions

Analytes in the standard solutions and in the samples were dissolved with a solution consisting of methanol:water 65:35 (v/v) [14,29]. Stock solutions of 1000 mg/kg were prepared for BA and SoA, by dissolving and diluting 10 mg of the pure reagents with the above solvent up to 10 mL. The same procedure was used to prepare the IS solution, but the only difference was that the standard compound was dissolved in pure methanol. The calibration solutions of BA (0.05, 0.1, 0.5, 1, 5, 10, 25, 50, and 100 mg/L) and SoA (0.25, 0.5, 1, 5, 10, 25, 50, and 100 mg/L) were prepared by diluting the stock solutions at appropriate ratios in the 10 mL volumetric flasks. It should be noted that these calibration ranges were within the instrument ranges. The working range of the method was determined by taking the dilution factor into account.

### 2.3. Samples and sample preparation

Six canned tomato paste samples, 6 tomato ketchup samples, 6 mayonnaise samples, and 6 barbeque sauce samples sold by different national and foreign brands were procured at local supermarkets. In addition, homemade samples including 3 tomato paste samples and 5 pepper paste samples that are sold openly were obtained from the local and open markets in İstanbul province, Türkiye in June 2023. The commercial samples were analyzed before the expiration dates.

A modified version of the initial sample preparation procedure was adapted from Nordic Committee on Food Analysis, NMKL method 124 [29]. Firstly, in order to minimize the waste volume of esterification reagents, namely sulfuric acid (H_2_SO_4_) and methanol (CH_3_OH), the sample weighing amount and the total solution volume were reduced. 0.2 g of a sample, of which stock sample had been thoroughly mixed and homogenized beforehand, was weighed and transferred accurately into a 10-mL volumetric flask with 3 mL of methanol:water 65:35 (v/v) solution. After the flask was vigorously shaken for 30 s, the solution was diluted up to 10 mL with methanol:water 65:35 (v/v) solution and the flask was shaken again. Thus, the samples were diluted approximately 50-fold. About 2–3 mL of each sample solution was filtered through filter paper.

**Figure 1:**
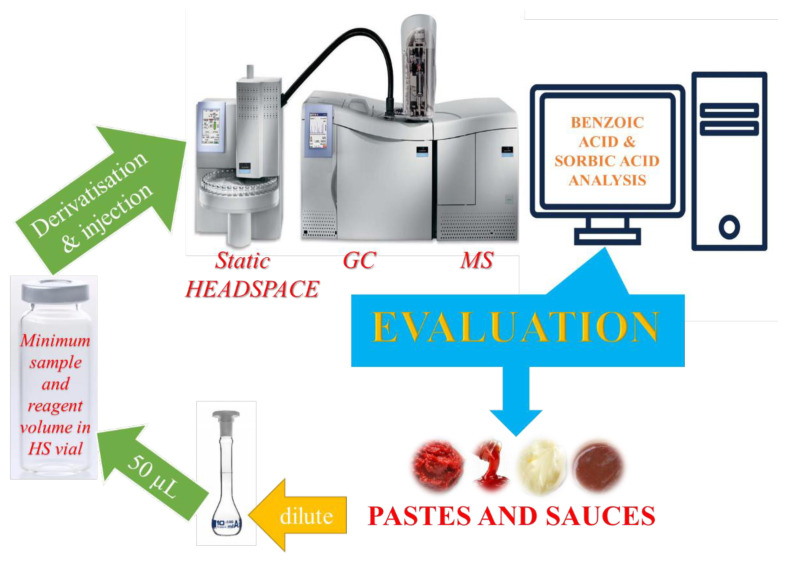
Graphical representation of simultaneous determination of BA and SoA in the pastes and sauces by using static HS-GC-MS.

From this point on, the HS parameters and the transferred volumes of the solutions previously optimized [14] for nonalcoholic beverages were utilized for incubation of the solutions in the HS vial, thereby derivatizing the analytes and the IS as their volatile methyl esters and subsequent on-line GC-MS analysis. Unlike this proposed study, 250 μL of the liquid samples examined in the previous study [14] was taken and diluted to 5 mL (DF = 20) with the same solvent mixture. In principle, a condensation reaction catalysed by sulfuric acid rapidly took place between the organic acids and methanol in a 22 mL HS vial. The reason why methanol was used in derivatization reaction was twofold; it was a low-boiling solvent in the preparation of solutions and also methyl esters boil at lower degrees when compared with other alcohols. What is more, no additional pure alcohol was not needed for the derivatization reaction. So, 50 μL of the 50-fold diluted sample solution (or water for blank solution), 50 μL of 10 mg/L IS, and 200 μL of 4.5 M sulfuric acid were mixed in a 22-mL HS vial. Then, the vials were crimped with the gas-tight Teflon-lined rubber septum caps. The frequency scanning shaker option of HS oven was activated during heating time, so equilibration time was reduced. After the incubation at 140 °C for 15 min, the samples from the gas phase were automatically injected to GC. A graphical summary of the HS-GC-MS analysis of BA and SoA in the samples is represented in Figure 1.

### 2.4. Instrumentation

Analyses were conducted by performing a Perkin Elmer Clarus 500 GC equipped with a Clarus 500 Mass Spectrometer and an HS40 autosampler. Perkin Elmer HS40 is a loopless headspace autosampler with an adjustable injection time. A polar phase (carbowax-PEG) Stabilwax-DA GC column (30 m length, 0.25 mm i.d., and 0.5 μm df.) used for chromatographic separations was taken from Restek.

The HS-GC-MS conditions optimized in the previous study [14] were exploited. The solutions in the 22 mL HS vials were heated in the HS oven at 140 °C during 15 min to complete derivatization and evaporation of the analytes and IS. The temperatures of the HS needle and the transfer line were adjusted to 145 and 150 °C, respectively. The vial pressurization, injection, and withdrawal time values were 1.00, 0.06, and 0.50 min, respectively. The flow rate of helium, employed as a carrier gas, was 1 mL/min and HS pressure was 30 psi. After injection, chromatographic separation was accomplished by setting the GC oven temperature was programmed as follows: initial, 100 °C for 6 min; ramp, 20 °C/min to 220 °C; and hold, at 220 °C for 3 min. GC injector temperature was 250 °C. The GC oven was equilibrated in 2 min and total analysis time was 15 min.

GC Inlet Line and Ion Source Temperature values were 200 °C and 180 °C, respectively. EI+ source energy was 70 eV. A TurboMass (version 6.1.0.1963) software was utilized for data acquisition and instrumental control for GC and MS by a computer. The data were acquired in the full scan mode, mass units were between 15–250 atomic mass units (amu) (4.1–12.0 min) and in the selected-ion recording (SIR) mode, *m/z* 67 for SoA (5.5–6.5 min), *m/z* 105 for BA (8.0–9.0 min), and *m/z* 120 for SaA (9.5–10.5 min) were simultaneously collected and processed by the TurboMass software. The peaks of the target analytes were identified and confirmed by both matching retention times of standards and performing MS spectral search program (NIST/EPA/NIH, ver 2.2.) on Total Ion Chromatogram (TIC) between 4.1–12.0 min. At the same time, BA and SoA were quantified by using SIR mode.

## 3. Results and discussion

In this work, a fast, accurate, and precise static headspace GC-MS method was suggested for different tomato paste, pepper paste, ketchup, mayonnaise, and barbecue sauce brands to determine the preservatives, namely benzoic acid (BA) and sorbic acid (SoA). BA and SoA were transferred from solid samples to the solutions during dilution with a 65:35 (v/v) solution of methanol and water. Secondly, they were derivatized by sulfuric acid/methanol in the static HS autosampler, in which their volatile methyl esters were formed. The last stage involved injection of the gaseous compounds into GC-MS system for analysis. The oven temperature, thermostat time, and injection time values of HS were very critical for derivatizing the organic acids and for establishing vapor pressure equilibrium of the compounds. GC oven temperature program parameters and also SIM and SIR modes of MS are important for the selectivity and sensitivity of the method. Therefore, the HS-GC-MS system parameters optimized in the study [14] for the analysis of BA and SoA in nonalcoholic beverages was adapted to this study.

### 3.1. Method validation study

Since the matrices of the samples examined in this study were different from the beverages, a full validation study was carried out. The linearity, sensitivity, precision, accuracy, and selectivity of the method were comprehensively investigated. All validation parameters are summarized in Table 1.

**Figure 2:**
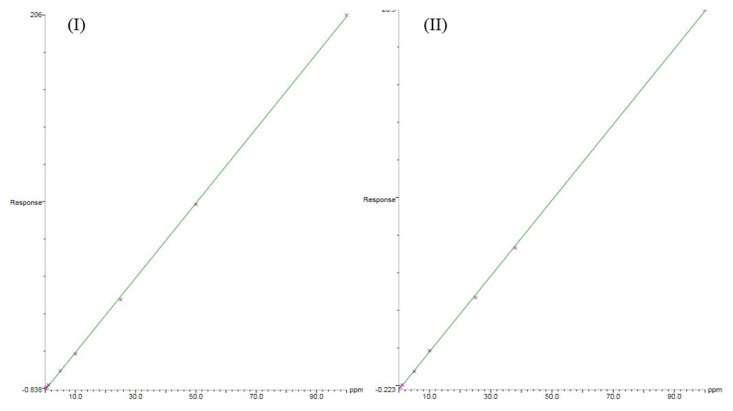
Calibration curves of BA (I) and SoA (II) provided by the TurboMass software in the report.

Linearity was assessed by internal standard calibration methods by using SalA as IS. The calibration curve of each analyte was constructed as a plot of the concentration of analyte (x axis) versus the ratio of area of analyte to IS (y axis). The calibration curves of BA and SoA which are shown in Figure 2 revealed that the measurements were conducted in the wide concentration ranges with very good linearities and correlations coefficients (r^2^ = 0.9998). The analytes and IS were successfully separated by using polar phase PEG column under the temperature program of the GC oven. The TurboMass software allowed processing SIR mode and full scan mode simultaneously. Thus, SIR mode was utilized for quantification while full scan mode was employed for the positive findings by scanning MS spectra in the NIST library. There was no interference observed at all, even with the samples with complex matrices, therefore one concludes that the proposed method is selective for the analytes in this study.

According to the Eurachem Guide [30], LODs and LOQs were calculated by 3 × SD and 10 × SD, respectively, of the average measurements (N = 10) of BA and SoA at low concentrations (S/N = 3). The sensitivity of this method was especially important for the paste samples, in which the use of preservatives is prohibited.

Precision was evaluated with RSD%_intraday_ (N = 6 injections) and RSD%_interday_ (N = 3 × 6 injections) values by analyzing the unbranded tomato paste sample No.1 (see also Table 2). The RSD% values that were found lower than 5 confirmed the method precision.

The accuracy was established by spiking an unbranded tomato paste sample and a mayonnaise sample containing 73% fat at three concentration levels. The recovery% values, which were in the range of 95%–105% for BA and 97%–104% for SoA (Table 1), were statistically evaluated by exerting the significance test at the 95% confidence level. According to Table 1, t_calculated_ < t_critical_ (2.57) was found for each result, which is indicative of the fact that the analytes were measured by the static HS-GC-MS method in the complex-matrixed samples with good accuracy that was statistically proven.

Since the vapor pressures of the volatile components are important in static HS studies, the matrices of the standards and samples in the HS vial should be similar. Before the optimized in-vial esterification reaction, the solid samples were sufficiently diluted in order to provide matrix matching with standard solutions. The successful results obtained from the method, which was fully validated after diluting liquid beverage samples 20-fold in the previous study [14], were achieved by diluting the samples 50-fold in this study. Accordingly, the working range and detection limit of the method, not the instrument, are presented in Table 1. Unlike GC or LC methods where injection is applied in liquid phase, in HS-GC methods, all matrix components except the VOCs could not reach the analytical column since injection is applied in the gas phase after heating in HS oven. At this point, it should be taken into consideration during heating in HS, which has a significant advantage in terms of eliminating the matrix effect, is that the partial vapor pressures of the analytes in the HS vial should be same for both standards and samples. Table 1 proves that in this study, by diluting the samples 50-fold, the partial vapor pressures of the analytes were compared to those of standard solutions and accordingly successful results were obtained. Consequently, very good precision and accuracy results were achieved.

### 3.2. Sample analysis

The author devised a sensitive, precise, and accurate HS-GC-MS method that was utilized for determination of BA and SoA in commercial branded samples consisting of 6 industrial canned tomato paste samples, 6 branded tomato ketchup samples, 6 branded barbeque sauce samples, and 6 branded mayonnaise samples, and also in unbranded, openly sold samples with traditional impression including 3 unbranded tomato paste samples and 5 unbranded pepper paste samples. Analyses were performed (N = 6) and the results were expressed as average ± standard deviation (mg/kg) in Table 2. Suitability of the results given in Table 2 were evaluated according to Turkish Food Codex Regulation on Food Additives [11] and Turkish Food Codex Communique on Tomato Paste and Similar Products [28].

**Figure 3:**
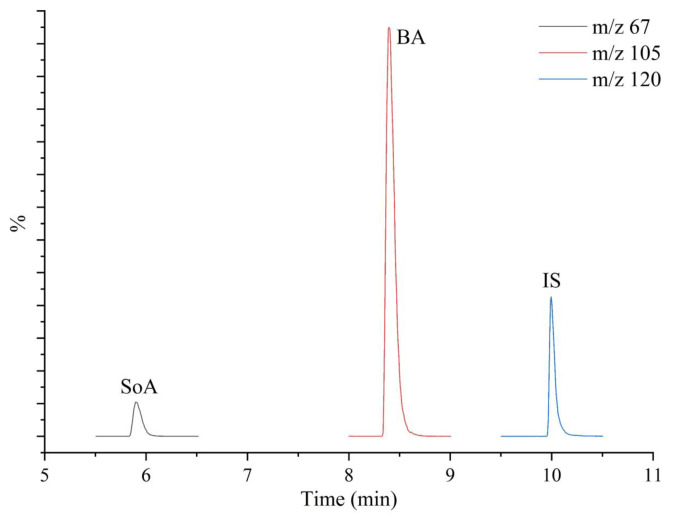
A representative SIR chromatogram of SoA (*m/z* 67), BA (*m/z* 105), and SalA (IS, *m/z* 120) obtained from HS-GC-MS analysis of the unbranded tomato paste sample (no 1).

As seen in Table 2, BA and SoA were not detected in any industrial canned tomato paste. Industrially known brands were found to comply with the regulations in tomato paste production, but unfortunately it was critical in unbranded products that were sold openly with a traditional impression. After the entry into force of this Turkish Food Codex Communiqué on Tomato Paste and Similar Products [28], the use of preservatives in pastes was prohibited. Previously, it had been stated in the Turkish Food Codex Regulation on Food Additives that only sorbic acid could be used in pastes and the maximum amount was 1000 mg/kg. Moreover, after the Communiqué came into force, products that were given a name or appearance similar to commercial pastes began to be seen, which could mislead the consumer. According to the communiqué, the labelling of food should not be misleading in terms of the qualities of the food. Accordingly, the brand, the name of the food, the expression, terms and visuals on the label of the food should not evoke another product group, especially in terms of the nature, identity, properties, composition, quantity, durability, country of origin and production method of the food. However, BA and SoA were determined both in the tomato pastes and in the plain and hot pepper pastes sold openly. Although these products must not contain any preservatives, the average total concentrations of preservatives were found to be quite high with > 2000 mg/kg in open tomato pastes, 764–2567 mg/kg in plain pepper pastes and even 1280 and 3145 mg/kg in a hot pepper paste samples (Table 2). The representative SIR chromatograms of an unbranded tomato paste sample (no. 1 in the 7th row of Table 2) is given in Figure 3. Even before the addition of preservatives in paste products was banned by the communique, only SoA had been allowed to be used, and the fact that even 500–1500 mg/kg BA was determined also in the samples analyzed in this study showed how risky the uncontrolled products sold openly.

Mayonnaise is an emulsified sauce and is at least partially based on an oil-in-water or a water-in-oil emulsion. According to Turkish Food Codex Regulation on Food Additives, BA and/or SoA can be added to mayonnaise alone or together. The maximum amount applies to the sum of these substances and these amounts are expressed in terms of free acid. If the fat content of a mayonnaise sample is 60% or more, the total concentration of BA and/or SoA must not exceed 1000 mg/kg, and if it is below 60%, the total concentration must not exceed 2000 mg/kg. In Table 2, the total preservative concentrations in mayonnaise sample no. 2 (73% fat) and sample no. 5 (68% fat) were found to be 831 and 774 mg/kg, respectively, while it was 1129.27 mg/kg for sample no. 4 (30% fat). On the other hand, neither BA nor SoA was detected in the mayonnaise samples with no. 1, 3, and 6. As a result, the information of preservative content declared on the labels of industrial well-known brand mayonnaise samples was verified and no mayonnaise product was found to be contrary to the regulation or misleading information on the label.

Tomato ketchup products are in the category of nonemulsified sauces. Thus, the maximum concentration limit of BA and/or SoA is 1000 mg/kg. Both preservatives were determined in ketchup samples numbered 4 and 6, which declared that they contain BA and SoA on their labels, and total preservative concentrations were found to be 332 and 475 mg/kg in two samples, respectively. BA and SoA were not detected in ketchup samples with no. 1, 2, 3, and 5 claiming to contain no preservatives on the label. In industrial ketchup samples, there was no violation of the regulations and communiqués in terms of preservatives. All of the barbeque sauce samples, which are another nonemulsified product type, included in this study were found to be suitable in terms of both the accuracy of the information declared on label and the BA and SoA concentrations.

### 3.3. Comparison of the proposed HS-GC-MS method with the other methods

Table 3 summarizes the comparison of the method proposed in this study with other methods selected from the literature. Noteworthy that Table 1 and Table 3 give the proposed method’s detection and quantification limits, but these values of the instrument were 0.03 and 0.09 mg/L for BA and 0.05 and 0.25 mg/L for SoA, respectively. In the study where BA and SoA determinations were carried out with CZE in mustard, ketchup, and tomato sauce samples [7], the important advantages are I) the sample preparation procedure does not contain solvents, ii) easy, and iii) the analysis is completed within 30 s. As seen in Table 2, chromatographic analyzes of BA and SoA take between 10–13 min. The proposed method can compete with other chromatographic methods in the literature in that its analysis time takes 11 min. However, the need to derivatize the analytes in this method is a disadvantage compared to other methods. On the other hand, the ability to perform quantitation in SIR mode and peak identification in TIC mode simultaneously is a great advantage and provides confidence by increasing the sensitivity and selectivity of the method.

## 4. Conclusion

In this study, BA and SoA contents of 32 samples including tomato paste, pepper paste, ketchup, mayonnaise, and barbeque sauce samples were examined by performing a validated static Headspace-GC-MS method. The solid sample could be analyzed directly with the HS, but by diluting the samples approximately 50 times with methanol:water 65:35 (v/v), both the methanol in the solvent was utilized in the in-vial derivatization reaction as alcohol at same time and the analyses were successfully conducted in the linear ranges. So, the most obvious advantages of the proposed method were I) sample preparation and derivatization were easy, fast, selective, not requiring costly clean-up procedure, producing minimal toxic chemical waste, which is a very important issue today, and II) the results are very reliable. In addition, the sensitivity of the proposed method was also an important parameter because the samples were diluted and detection of BA and SoA was important especially in tomato and pepper pastes in which the preservatives were prohibited. The results obtained from the real samples revealed that the known branded commercial samples obeyed the legal limits whereas BA and SoA were determined in the tomato and pepper pastes sold openly. Unfortunately, some additives and many substances for fraudulent purposes can be put into tomato and pepper pastes unconsciously and disproportionately, which are usually produced in unhygienic conditions and sold openly under the traditional or homemade name, accordingly public health is endangered. In addition to the use of additives, the other paste-based products should be in such a way that they do not create the impression of tomato or pepper paste on the consumer. Noteworthy is that the pastes that are regularly inspected and produced in a controlled manner in hygienic conditions should be preferred instead of the open products. Therefore, the control of counterfeiting and adulteration in unbranded tomato and pepper pastes, which are widely consumed across the globe, and also the reliability of the methods used for analysis are of great importance. Consequently, the proposed HS-GC-MS method can be applied to any type of sauce sample for food quality control.

## Figures and Tables

**Table 1:** Validation parameters of the proposed method.

Parameter		BA			SoA		
Linearity	Calibration range (mg/L)	2.5–5000			12.5–5000		
	Correlation coefficient (R^2^)	0.9998			0.9998		
	Regression equation	*y* = 2.0602*x* – 0.4273			*y* = 0.2060*x* – 0.0258		
Selectivity	Selected ion (m/z)	105			67		
	Retention time (min)	8.39			5.89		
		No interference			No interference		
		*(Verified with the measurements of all real samples)*					
Sensitivity (in the sample)	LOD (mg/kg)	1.5			4.5		
	LOQ (mg/kg)	2.5			12.5		
Intra-day Precision (RSD%)	(N = 6 measurements of unbranded open tomato paste sample (No 1))	2.8			4.1		
Interday Precision (RSD%)	(N = 3 × 6 measurements of unbranded open tomato paste sample (No 1))	4.3			4.7		
Accuracy	Open tomato paste sample (No 1)						
	Initial (mg/kg) (N = 6)	1551 ± 31			606 ± 15		
	Added (mg/kg)	750	1500	3000	300	600	1200
	Found (mg/kg) (N = 6)	2326 ± 102	2996 ± 87	4712 ± 198	916 ± 33	1213 ± 55	1803 ± 88
	Recovery%	96.7 ± 4.4	103.8 ± 2.9	94.9 ± 4.2	96.9 ± 3.6	98.8 ± 4.5	100.3 ± 4.9
	t_cal_	1.30	2.27	2.10	1.49	0.46	0.11
	Mayonnaise sample (No 2)						
	Initial (mg/kg) (N = 6)	345 ± 10			485 ± 23		
	Added (mg/kg)	150	300	600	250	500	1000
	Found (mg/kg) (N = 6)	503 ± 20	630 ± 26	970 ± 43	725 ± 27	988 ± 26	1515 ± 50
	Recovery%	94.8 ± 3.9	105.4 ± 4.1	96.0 ± 4.4	104.2 ± 3.7	99.5 ± 2.6	97.1 ± 3.3
	t_cal_	2.31	2.28	1.57	1.97	0.33	1.52

**Table 2:** The results of SoA and BA concentrations in tomato pastes, pepper pastes, ketchups, mayonnaises and barbeques determined by the proposed HS-GC-MS method (N = 6).

No	Sample	SoA		BA	

		mg/kg	Declared on the label	mg/kg	Declared on the label
1	Industrial canned tomato paste 1	< LOQ	not contain	< LOQ	not contain
2	Industrial canned tomato paste 2	< LOQ	not contain	< LOQ	not contain
3	Industrial canned tomato paste 3	< LOQ	not contain	< LOQ	not contain
4	Industrial canned tomato paste 4	< LOQ	not contain	< LOQ	not contain
5	Industrial canned tomato paste 5	< LOQ	not contain	< LOQ	not contain
6	Industrial canned tomato paste 6	< LOQ	not contain	< LOQ	not contain
7	Unbranded tomato paste (open) 1	1551 ± 31	no label	606 ± 15	no label
8	Unbranded tomato paste (open) 2	1378 ± 54	no label	637 ± 32	no label
9	Unbranded tomato paste (open) 3	1412 ± 40	no label	562 ± 21	no label
10	Unbranded plain pepper paste (open) 1	906 ± 32	no label	638 ± 26	no label
11	Unbranded plain pepper paste (open) 2	505 ± 11	no label	258 ± 14	no label
12	Unbranded plain pepper paste (open) 3	1095 ± 16	no label	1472 ± 37	no label
13	Unbranded hot pepper paste (open) 1	1144 ± 37	no label	135 ± 8	no label
14	Unbranded hot pepper paste (open) 2	1318 ± 26	no label	1827 ± 46	no label
15	Industrial tomato ketchup 1	< LOQ	not contain	< LOQ	not contain
16	Industrial tomato ketchup 2	< LOQ	not contain	< LOQ	not contain
17	Industrial tomato ketchup 3	< LOQ	not contain	< LOQ	not contain
18	Industrial tomato ketchup 4	137 ± 5	contains	195 ± 11	contains
19	Industrial tomato ketchup 5	< LOQ	not contain	< LOQ	not contain
20	Industrial tomato ketchup 6	221 ± 9	contains	253 ± 6	contains
21	Industrial mayonnaise 1	< LOQ	undeclared	< LOQ	undeclared
22	Industrial mayonnaise 2	345 ± 10	contains	485 ± 23	contains
23	Industrial mayonnaise 3	< LOQ	undeclared	< LOQ	undeclared
24	Industrial mayonnaise 4	993 ± 29	contains	137 ± 5	contains
25	Industrial mayonnaise 5	< LOQ	undeclared	774 ± 23	contains
26	Industrial mayonnaise 6	< LOQ	not contain	< LOQ	not contain
27	Industrial barbeque sauce 1	595 ± 16	contains	110 ± 6	contains
28	Industrial barbeque sauce 2	148 ± 4	undeclared	146 ± 8	undeclared
29	Industrial barbeque sauce 3	347 ± 13	contains	188 ± 4	contains
30	Industrial barbeque sauce 4	< LOQ	undeclared	430 ± 19	contains
31	Industrial barbeque sauce 5	< LOQ	not contain	< LOQ	not contain
32	Industrial barbeque sauce 6	< LOQ	not contain	< LOQ	not contain

**Table 3:** Comparison of the proposed HS-GC-MS method with the other methods selected in the literature.

Matrix	Sample Preparation	Analytical Instrument	Analysis time a	Analyte	LOD (mg/L)	LOQ (mg/L)	Reference
Mustard, Ketchup, Tomato Sauce	Aqueous dilution, centrifugation, filtration	CZE	30 s	BA	0.9	1.3	[7]
				SoA	0.3	1.1	
Foodstuffs	aqueous dilution, DLLME	HPLC-UV	10 min	BA	0.1	0.5	[31]
				SoA	0.08	0.3	
beverages and soy sauce	air-assisted DLLME procedure with organic phase solidification	HPLC-UV	13 min	BA	0.03	0.1	[8]
				SoA	0.02	0.5	
Soy sauce	Off-line liquid-phase extraction (LPE)	FESI-CE-C^4^D^b^	10 min	BA	0.01	0.03^c^	[32]
				SoA	0.006		
Fermented shrimp paste, pickled vegetable, soy sauce, fish sauce.	HS-SPME	GC-FID	12 min	BA	1.4	4.2	[33]
				SoA	1.1	3.2	
tomato paste, pepper paste, ketchup, mayonnaise, and barbeque sauce	dilution, filtration, in-vial derivatization in HS	HS-GC-MS	11 min	BA	1.5	4.5	This study
				SoA	2.5	12.5	

a Total analysis times declared in the study or read from chromatograms and electropherograms.

b CE using field-amplified sample injection with capacitively coupled contactless conductivity detection.

c LOQ values are not given, the lowest concentrations of the calibration range for the two analytes are presented as the same.

## References

[1] Zengin N Yüzbaşioĝlu D Ünal F Yilmaz S Aksoy H The evaluation of the genotoxicity of two food preservatives: Sodium benzoate and potassium benzoate Food and Chemical Toxicology 2011 49 4 763 9 10.1016/j.fct.2010.11.040 21130826

[2] Hsu SH Hu CC Chiu TC Online dynamic pH junction-sweeping for the determination of benzoic and sorbic acids in food products by capillary electrophoresis Analytical and Bioanalytical Chemistry 2014 406 2 635 41 10.1007/s00216-013-7481-1 24247553

[3] Junqueira De Carvalho L Cristina Pires Do Rego E Carius Garrido B Quantification of benzoic acid in beverages: The evaluation and validation of direct measurement techniques using mass spectrometry Analytical Methods 2016 8 14 2955 60 10.1039/c5ay03168k

[4] Bian Y Wang Y Yu J Zheng S Qin F Analysis of six preservatives in beverages using hydrophilic deep eutectic solvent as disperser in dispersive liquid-liquid microextraction based on the solidification of floating organic droplet Journal of Pharmaceutical and Biomedical Analysis 2021 195 113889 10.1016/j.jpba.2021.113889 33429250

[5] D’Amore T Di Taranto A Berardi G Vita V Iammarino M Going green in food analysis: A rapid and accurate method for the determination of sorbic acid and benzoic acid in foods by capillary ion chromatography with conductivity detection Lwt 2021 141 November 2020 110841 10.1016/j.lwt.2020.110841

[6] Feng J Li J Huang W Cheng H Zhang Z Capillary Zone Electrophoresis Determination of Five Trace Food Additives in Beverage Samples Using Counterflow Transient Isotachophoresis Food Analytical Methods 2021 14 2 380 8 10.1007/s12161-020-01894-1

[7] Pereira LM Della Betta F Seraglio SKT Schulz M Nehring P Assessment of sorbate and benzoate content in mustard, ketchup and tomato sauce by sub-minute capillary electrophoresis Food Technology and Biotechnology 2021 59 3 376 84 10.17113/ftb.59.03.21.7095 34759768 PMC8542181

[8] Timofeeva I Kanashina D Stepanova K Bulatov A A simple and highly-available microextraction of benzoic and sorbic acids in beverages and soy sauce samples for high performance liquid chromatography with ultraviolet detection Journal of Chromatography A 2019 1588 1 7 10.1016/j.chroma.2018.12.030 30579637

[9] The European Parliament and the Council of the European Union Regulation (EU) 1333/2008 The Official Journal of the European Union 2008 1333 48 119

[10] European Commission Regulation (EC) No 1333/2008 of the European Parliament and of the Council of 16 December 2008 on food additives The Official Journal of the European Union 2008

[11] The Turkish Food Codex Regulation on food additives 2013 [cited 2023 Aug 30].

[12] Dong C Wang W Headspace solid-phase microextraction applied to the simultaneous determination of sorbic and benzoic acids in beverages Analytica Chimica Acta 2006 562 1 23 9 10.1016/j.aca.2006.01.045

[13] Sagandykova GN Alimzhanova MB Nurzhanova YT Kenessov B Determination of semi-volatile additives in wines using SPME and GC–MS Food Chemistry 2017 220 162 7 10.1016/j.foodchem.2016.09.164 27855884

[14] Destanoğlu O Simultaneous determination of benzoic acid and sorbic acid in non-alcoholic beverages by a validated HS-GC-MS method with reduced waste Food Additives & Contaminants: Part A 2023 40 7 812 23 10.1080/19440049.2023.2224891 37326484

[15] Techakriengkrai I Surakarnkul R Analysis of benzoic acid and sorbic acid in Thai rice wines and distillates by solid-phase sorbent extraction and high-performance liquid chromatography Journal of Food Composition and Analysis 2007 20 3–4 220 5 10.1016/j.jfca.2006.10.003

[16] Ozer H Psimouli V Ozcan N Ozer B Papadaki I Ring trial for the simultaneous analysis of sweeteners and preservatives in soft drinks Quality Assurance and Safety of Crops and Foods 2013 5 1 71 7 10.3920/QAS2012.0124

[17] Zhao YG Chen XH Yao SS Pan SD Li XP Analysis of nine food additives in red wine by ion-suppression reversed-phase high-performance liquid chromatography using trifluoroacetic acid and ammonium acetate as ion-suppressors Analytical Sciences 2012 28 10 967 71 10.2116/analsci.28.967 23059992

[18] Imanulkhan Setyaningsih W Rohman A Palma M Development and validation of hplc-dad method for simultaneous determination of seven food additives and caffeine in powdered drinks Foods 2020 9 8 1 12 10.3390/foods9081119 PMC7466259 32823790

[19] Xu J Chen B He M Hu B Analysis of preservatives with different polarities in beverage samples by dual-phase dual stir bar sorptive extraction combined with high-performance liquid chromatography Journal of Chromatography A 2013 1278 8 15 10.1016/j.chroma.2012.12.061 23336943

[20] Sugiura J Nakajima M Simultaneous determination of nine preservatives in food by liquid chromatography with the aid of coagulant in the clean-up process Food Additives and Contaminants - Part A Chemistry, Analysis, Control, Exposure and Risk Assessment 2017 34 5 695 704 10.1080/19440049.2017.1293302 28277177

[21] Iwakoshi K Shiozawa Y Yamajima Y Baba I Monma K Determination of nine preservatives in processed foods using a modified QuEChERS extraction and quantified by HPLC-PDA Food Additives and Contaminants - Part A Chemistry, Analysis, Control, Exposure and Risk Assessment 2019 36 7 1020 31 10.1080/19440049.2019.1615644 31100042

[22] Ulca P Atamer B Keskin M Senyuva HZ Sorbate and benzoate in Turkish retail foodstuffs Food Additives and Contaminants: Part B Surveillance 2013 6 3 209 13 10.1080/19393210.2013.795609 24779907

[23] Ochiai N Sasamoto K Takino M Yamashita S Daishima S Simultaneous determination of preservatives in beverages, vinegar, aqueous sauces, and quasi-drug drinks by stir-bar sorptive extraction (SBSE) and thermal desorption GC-MS Analytical and Bioanalytical Chemistry 2002 373 1–2 56 63 10.1007/s00216-002-1257-3 12012172

[24] Mato I Huidobro JF Simal Lozano J Sancho MT Simultaneous determination of organic acids in beverages by capillary zone electrophoresis Analytica Chimica Acta 2006 565 2 190 7 10.1016/j.aca.2006.02.043

[25] Zhang X Xu S Sun Y Wang Y Wang C Simultaneous determination of benzoic acid and sorbic acid in food products by CE after on-line preconcentration by dynamic pH junction Chromatographia 2011 73 11–12 1217 21 10.1007/s10337-011-2009-3

[26] Yu M Wen R Jiang L Huang S Fang Z Rapid analysis of benzoic acid and vitamin C in beverages by paper spray mass spectrometry Food Chemistry 2018 268 June 2017 411 5 10.1016/j.foodchem.2018.06.103 30064777

[27] Ohtsuki T Sato K Sugimoto N Akiyama H Kawamura Y Absolute quantitative analysis for sorbic acid in processed foods using proton nuclear magnetic resonance spectroscopy Analytica Chimica Acta 2012 734 54 61 10.1016/j.aca.2012.04.033 22704472

[28] Turkish Food Codex Communique on Paste and Similar Products 2021 [cited 2023 Aug 30].

[29] Nordic Committee on Food Analysis N method 124 Benzoic acid, sorbic acid and p-hydroxybenzoic acid esters Liquid Chromatographic Determination in Foods 1997

[30] Eurachem The Fitness for Purpose of Analytical Methods; A Laboratory Guide to Method Validation and Related Topics 2nd ed 2014 [cited 2023 Aug 30]

[31] Javanmardi F Nemati M Ansarin M Arefhosseini SR Benzoic and sorbic acid in soft drink, milk, ketchup sauce and bread by dispersive liquid–liquid microextraction coupled with HPLC Food Additives and Contaminants: Part B Surveillance 2015 8 1 32 9 10.1080/19393210.2014.955534 25135626

[32] Wei R Li W Yang L Jiang Y Xie T Online preconcentration in capillary electrophoresis with contactless conductivity detection for sensitive determination of sorbic and benzoic acids in soy sauce Talanta 2011 83 5 1487 90 10.1016/j.talanta.2010.11.036 21238741

[33] Tungkijanansin N Alahmad W Nhujak T Varanusupakul P Simultaneous determination of benzoic acid, sorbic acid, and propionic acid in fermented food by headspace solid-phase microextraction followed by GC-FID Food Chemistry 2020 329 April 127161 10.1016/j.foodchem.2020.127161 32502744

